# Addiction and chronic skin diseases: A Pan‐European study on prevalence, associations and patient impact

**DOI:** 10.1111/jdv.70245

**Published:** 2025-12-14

**Authors:** Stefanie Ziehfreund, Magdalena Saak, Alisa Schaal, Roxana Mazilu, Emmanuel Mahé, Carla Hajj, Emma K. Johansson, Josefin Lysell, Gunnthorunn Sigurdardottir, Franz J. Legat, Catherina Koch, Astrid Schmieder, Caroline Glatzel, Brian Kirby, Emily Pender, Anna Zalewska‐Janowska, Katarzyna Tomaszewska, Giulia Ciccarese, Pia Lauriola, Michael J. Boffa, Stephanie Farrugia, Anna Caroline Pilz, Franziska Schauer, Magdalena Trzeciak, Weronika Zysk, Krisztián Gáspár, Andrea Szegedi, Giovanni Damiani, Andrea Carugno, Ilona Hartmane, Ingmars Mikazans, Skaidra Valiukeviciene, Livija Petrokaite, Jacek C. Szepietowski, Beata Jastrząb, Petra Staubach, Suzana Ljubojević Hadžavdić, Daška Štulhofer Buzina, Christian Posch, Jakob Jochmann, Antonia Wiala, Ludwig Scheffenbichler, Emanuele Trovato, Laura Calabrese, Gyula Laszlo Fekete, Pavel Chernyshov, Lidiia Kolodzinska, Julia Welzel, Karisa Thölken, Julia‐Tatjana Maul, Lara Valeska Maul, Damian Meyersburg, Stephanie Bowe, Cathal O. Connor, Razvigor Darlenski, Stephan Traidl, Christian Vestergaard, Elena Lucía Pinto‐Pulido, Maria Polina Konstantinou, Eszter Szlávicz, Kristine Heidemeyer, Nikhil Yawalkar, Hanka Agnes Caroline Lantzsch, Aleksandra Soplinska, Anna‐Theresa Seitz, Tiago Torres, Zenon Brzoza, Ana Sanader Vučemilović, Liana Manolache, Nina Magnolo, Emanuele Scala, Kilian Eyerich, Tilo Biedermann, Alexander Zink

**Affiliations:** ^1^ Department of Dermatology and Allergy, TUM School of Medicine and Health Technical University of Munich Munich Germany; ^2^ Dermatology Department, Centre Hospitalier Victor Dupouy Argenteuil Cedex France; ^3^ Division of Dermatology and Venereology, Department of Medicine Solna Karolinska Institutet Stockholm Sweden; ^4^ Department of Dermatology and Venereology Karolinska University Hospital Stockholm Sweden; ^5^ Department of Dermatology and Venereology in Östergötland Linköping University Linköping Sweden; ^6^ Department of Biomedical and Clinical Sciences Linköping University Linköping Sweden; ^7^ Department of Dermatology Medical University of Graz Graz Austria; ^8^ Das Kurhaus Bad Gleichenberg Bad Gleichenberg Austria; ^9^ Department of Dermatology, Venereology and Allergology University Hospital Würzburg Wurzburg Germany; ^10^ Charles Department of Dermatology, St. Vincent's University Hospital Dublin Ireland; ^11^ Psychodermatology and Neuroimmunobiology of Skin Department Medical University of Lodz Łódź Poland; ^12^ Unit of Dermatology, Department of Medical and Surgical Sciences University of Foggia Foggia Italy; ^13^ Department of Dermatology, Mater Dei Hospital Msida Malta; ^14^ Department of Dermatology, Medical Center – University of Freiburg, Faculty of Medicine University of Freiburg Freiburg Germany; ^15^ Department of Dermatology, Venereology and Allergology, Faculty of Medicine Medical University of Gdansk Gdansk Poland; ^16^ Department of Dermatology, Venereology and Allergology University Clinical Center Gdansk Poland; ^17^ Department of Dermatology, Faculty of Medicine University of Debrecen Debrecen Hungary; ^18^ Department of Biomedical, Surgical, and Dental Sciences University of Milan Milan Italy; ^19^ Italian Centre for Coordinated Research in Precision Medicine and Chronic Inflammation University of Milan Milan Italy; ^20^ Dermatology Unit, Department of Medicine and Surgery University of Insubria Varese Italy; ^21^ Department of Dermatology and Venerology Riga Stradinš University Riga Latvia; ^22^ Department of Skin and Venereal Diseases Lithuanian University of Health Sciences Kaunas Lithuania; ^23^ Department of Dermato‐Venerology, 4th Military Hospital Wroclaw Poland; ^24^ Faculty of Medicine Wroclaw University of Science and Technology Wroclaw Poland; ^25^ University Centre of General Dermatology and Oncodermatology Wroclaw Medical University Wroclaw Poland; ^26^ Department of Dermatology University Medical Center Mainz Mainz Germany; ^27^ Department of Dermatology and Venereology University Hospital Center Zagreb Zagreb Croatia; ^28^ Department for Dermatology, Clinic Hietzing Vienna Healthcare Group Vienna Austria; ^29^ School of Medicine Sigmund Freud University Vienna Austria; ^30^ Department for Dermatology and Venereology, Clinic Landstrasse Vienna Healthcare Group Vienna Austria; ^31^ Department for Dermatology and Venereology, Clinic Donaustadt Vienna Healthcare Group Vienna Austria; ^32^ Dermatology Unit, Department of Medical, Surgical and Neurological Sciences University of Siena Siena Italy; ^33^ Dermatology Department, ‘George Emil Palade’ University of Medicine, Pharmacy, Science and Technology Targu Mures Romania; ^34^ CMI Dermamed Private Medical Office Targu Mures Romania; ^35^ Department of Dermatology and Venereology National Medical University Kiev Ukraine; ^36^ Khmelnytskyi City Medical and Diagnostic Center, 2nd Polyclinic Khmelnytskyi Ukraine; ^37^ Department of Dermatology and Allergology Augsburg University Hospital Augsburg Germany; ^38^ Faculty of Medicine University of Zürich Zurich Switzerland; ^39^ Department of Dermatology University Hospital of Zurich Zurich Switzerland; ^40^ Department Dermatology and Venereology University Hospital of Zurich Zurich Switzerland; ^41^ Department of Dermatology and Allergology University Hospital Salzburg of the Paracelsus Medical University Salzburg Austria; ^42^ Department of Dermatology, South Infirmary Victoria Hospital Cork Ireland; ^43^ Department of Dermatology and Venereology Trakia University Stara Zagora Bulgaria; ^44^ Department of Dermatology and Allergy Hannover Medical School Hannover Germany; ^45^ Department of Dermatology Aarhus University Hospital Aarhus Denmark; ^46^ Department of Dermatology, Hospital Universitario Príncipe de Asturias Madrid Spain; ^47^ Department of Dermatology University General Hospital of Heraklion Heraklion Greece; ^48^ Department of Dermatology, Venereology and Oncodermatology, Medical Faculty University of Pécs Pécs Hungary; ^49^ Archiepiscopal College of Veszprém Veszprém Hungary; ^50^ Department of Dermatology Bern University Hospital, University of Bern Inselspital Switzerland; ^51^ Department of Dermatology and Allergology Asklepios Nordseeklinik Sylt/Westerland Sylt OT Westerland Germany; ^52^ Department of Dermatology Medical University of Warsaw Warsaw Poland; ^53^ Department of Dermatology, Venerology and Allergology, Faculty of Medicine Leipzig University Leipzig Germany; ^54^ Department of Dermatology, Centro Hospitalar Universitário de Santo António University of Porto Porto Portugal; ^55^ Department of Internal Diseases with Division of Allergology University of Opole Opole Poland; ^56^ Department of Dermatovenerology University Hospital Centre Split Split Croatia; ^57^ Dermatology, Dali Medical Bucharest Romania; ^58^ Department of Dermatology University Hospital of Muenster Münster Germany; ^59^ Laboratory of Experimental Immunology, Istituto Dermopatico dell'Immacolata‐Istituto di Ricovero e Cura a Carattere Scientifico (IDI‐IRCCS) Rome Italy

**Keywords:** alcohol use, addictive behaviour, atopic dermatitis, comorbidity, cross‐sectional study, Europe, gambling, Hidradenitis Suppurativa, internet addiction, patient‐reported outcomes, psoriasis, quality of life, skin diseases, stigmatization, urticaria, Vitiligo

## Abstract

**Background:**

Chronic skin diseases such as psoriasis (PSO), atopic dermatitis (AD) and hidradenitis suppurativa (HS) are frequently associated with psychological distress, potentially promoting maladaptive coping mechanisms including addictive behaviours. Despite evidence of higher addiction rates among dermatology patients, comprehensive multicenter data across Europe are lacking.

**Objectives:**

To estimate the prevalence and patterns of addictive behaviours among patients with chronic skin diseases in European tertiary dermatology centres and explore associated sociodemographic and clinical factors.

**Methods:**

This multicentre cross‐sectional study recruited adult patients with PSO, AD, HS, alopecia areata (AA), urticaria, or vitiligo from dermatology departments in 20 European countries. Participants completed a standardized questionnaire assessing sociodemographics, disease characteristics, and addictive behaviours (smoking, alcohol use, drug use, gambling, internet addiction, and eating disorders). Descriptive analyses and multivariate logistic regression were performed.

**Results:**

Among 3585 participants (median age 43 years; 51.1% female), the prevalence of addictive behaviours was notable: smoking (25.7%), pathological gambling (4.5%), hazardous drinking (8.8%), alcohol dependence (2.5%), drug use disorders (5.3%), eating disorders (1.8%), and internet addiction (29.7%). Smoking was most common among PSO and HS patients (48.6%), and gambling among AA and vitiligo patients (8.2%). Significant associations included male sex, younger age, single status, higher Dermatology Life Quality Index (DLQI) scores, and regional variation.

**Discussion:**

Addictive behaviours are prevalent in dermatology patients and are associated with both sociodemographic and disease‐related factors. The DLQI was positively correlated with multiple addictions, suggesting that reduced quality of life may contribute to maladaptive coping. However, due to the absence of a control group, the tertiary care setting, limited center distribution, and unknown response rate, generalizability is restricted.

**Conclusions:**

Addiction screening and supportive mental health strategies should be integrated into dermatologic care, particularly for high‐risk patients. Population‐based studies with control groups are needed to confirm these findings.

## Plain Language Summary

**What is the disease?:**

Chronic skin diseases such as psoriasis, atopic dermatitis, hidradenitis suppurativa, urticaria, alopecia areata and vitiligo are long‐lasting conditions that can cause visible symptoms, emotional distress and social stigma. Many people with these diseases also experience mental health challenges.

**Location of the study:**

This study was carried out in dermatology departments across 20 European countries, involving a large number of hospitals and university centres.

**Aim of the study:**

The researchers wanted to find out how common addictive behaviours—such as smoking, gambling, alcohol and drug use, eating disorders and internet addiction—are among people with chronic skin diseases and which factors are linked to them.

**Methodology:**

A total of 3585 adults with one of six chronic skin diseases completed a detailed questionnaire about their lifestyle, skin condition and quality of life. The study looked for patterns and associations between these factors and different forms of addiction.

**Key findings:**

Addictive behaviours were common: about *one in four smoked*, *one in three showed signs of internet addiction* and *nearly one in 10* reported risky alcohol use. Men, younger people and those who were single or had a lower quality of life were more likely to have an addiction. Rates also varied between European regions.

**Conclusions and practical implications:**

Addictions are frequent among people with chronic skin diseases. Screening for addictive behaviours and offering mental health support should become a routine part of dermatologic care to improve overall well‐being and treatment success.

## Graphical abstract

This pan‐European study reveals high rates of addiction in patients with chronic skin diseases. Smoking, alcohol, drug use, gambling, and internet addiction were prevalent and associated with quality of life, demographics, and region. The findings support integrating addiction screening into dermatologic care to improve outcomes.

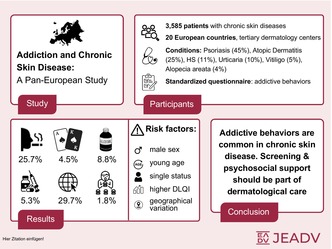

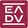

**Why was the study undertaken?**

- To explore the prevalence of addictive behaviours among patients with chronic skin diseases across Europe and identify demographic and disease‐specific factors associated with addiction.

**What does this study add?**

- This study provides comprehensive data on addiction comorbidity in tertiary dermatology centres, revealing significant variations in addiction prevalence across skin conditions and European regions and highlighting associations such as age, gender, relationship status and quality of life (DLQI).

**What are the implications of this study for disease understanding and/or clinical care?**

- Integrating addiction screening into dermatologic care could enhance patient outcomes, addressing both physical and psychological needs and informing targeted, region‐specific interventions.

## INTRODUCTION

Chronic skin diseases, including psoriasis (PSO), atopic dermatitis (AD), hidradenitis suppurativa (HS), alopecia areata, urticaria and vitiligo, affect a significant portion of the global population.^1^, ^2^ Today, these diseases are seen as systemic disorders^3^ with numerous associated comorbidities beyond their visible skin findings.^4^, ^5^, ^6^ Patients suffering from skin diseases face a range of psychological challenges, including depression, anxiety, social stigma, diminished self‐esteem, and reduced quality of life.^4^, ^5^, ^6^ While advances in understanding the inflammatory and genetic underpinnings of skin diseases have led to effective therapies, the psychological burden remains a significant and less addressed issue, particularly in relation to mental health.

The psychological burden of living with dermatologic conditions is seen with an increased vulnerability to addictive behaviours as coping mechanisms.^7^, ^8^ Recent research further shows a notable overlap between chronic skin diseases and addictive behaviours, such as smoking, alcohol, and drug abuse, internet addiction and pathological gambling.^9^, ^10^ The bidirectional relationship between skin conditions and addiction is complex, involving both psychological and biological factors but is not yet fully understood.^8^, ^11^, ^12^, ^13^ For example, smoking and alcohol use not only serve as coping mechanisms but may also exacerbate inflammatory pathways, worsening the course of these diseases and reducing treatment efficacy.^14^ Overall, patients with chronic skin conditions demonstrate higher rates of addiction than the general population, with alcohol being particularly prevalent in PSO.^15^, ^16^

Addiction and substance use disorders can significantly impair treatment outcomes in chronic skin diseases by reducing adherence to dermatologic therapies and exacerbating symptoms, as individuals may prioritize substance abuse over medical regimens.^17^ This non‐compliance can worsen symptoms and disease severity over time. Behavioural and cognitive impairments associated with addiction—such as poor decision‐making and reduced impulse control—further complicate the management of skin conditions requiring consistent treatment.^18^ Additionally, psychological stress common in those with substance use disorders can intensify inflammatory responses, aggravating conditions like PSO and AD.^19^ The interplay between stress and addiction may worsen both physiological and behavioural outcomes, complicating care for patients with comorbid dermatologic and addiction issues.^20^ Moreover, substance abuse often exacerbates mental health issues and social isolation, further hindering effective treatment.^20^, ^21^

Previous studies have typically examined single skin conditions or specific addictive behaviours, offering limited insight into how addiction prevalence varies across different skin diseases and regions. This gap is further influenced by sociodemographic factors such as age, gender, employment, and relationship status, which are known to affect addiction susceptibility.^22^ Addiction prevalence varies across countries, and among patients with chronic skin conditions, men generally show higher overall addiction rates.^23^, ^24^, ^25^

This study investigates the prevalence and patterns of addictive behaviours among patients with chronic skin diseases in tertiary dermatology centres across 20 European countries. By analysing addictions alongside demographic, regional and disease‐specific factors, it aims to deepen understanding of addiction comorbidities in dermatology and support the integration of addiction screening and management into patient‐centred care.

## METHODS

### Study design

A cross‐sectional multi‐centre study was conducted within dermatology departments in 20 European countries (Table S1). Patients with diagnosed alopecia areata, AD, hidradenitis suppurativa (HS), psoriasis, urticaria and vitiligo were recruited consecutively from 01 July 2023 to 31 July 2024. Recruitment was conducted either during outpatient visits or through follow‐up contact, depending on local logistics and ethical requirements. Importantly, patients were not pre‐identified via electronic health records or other databases. Instead, inclusion was based on confirmed clinical diagnosis of one of the six target dermatological conditions by a dermatologist at the time of participation, ensuring diagnostic accuracy and minimizing selection bias. Participants completed a standardized questionnaire either online or on paper in their native languages. Inclusion criteria were a clinically confirmed diagnosis of any of the abovementioned chronic skin diseases and being ≥18 years old. Exclusions were for local language barriers and individuals affected by more than one of the chronic diseases of interest. Written informed consent was obtained from all participants before inclusion, and ethical adherence was ensured, with approvals from all participating centres, including the lead ethical committee at the Medical Faculty at Technical University of Munich, Germany (reference 2023‐308‐S‐KH).

### Questionnaire

Paper‐based questionnaires were inputted into REDCap (Research Electronic Data Capture, Vanderbilt University), which was also utilized for online submissions.

Sociodemographic parameters included year of birth, gender (‘male’, ‘female’, ‘diverse’), residence (‘urban’, ‘rural’), weight (kg), height (cm), employment (‘employed’, ‘unemployed’) and marital status (‘single’, ‘in a relationship’, ‘married’, ‘divorced/separated’, ‘widowed’).

Disease‐related parameters were asked for regarding the skin condition, the year of the initial diagnosis and subjective perceived disease severity on an 11‐point scale ranging from 0 to10 (‘not severely affected’ to ‘severely affected’). Disease‐related QoL was measured using the DLQI, comprising 10 items, each rated from 0 to 3 with total scores ranging from 0 to 30, with higher scores on the DLQI indicating greater impairment.^26^ Heuristic happiness was assessed using a single item on an 11‐point Likert scale (ranging from 0 = ‘extremely unhappy’ to 10 = ‘extremely happy’), adapted from the European Social Survey.^27^

Tobacco use was assessed by asking, ‘How many/how often do you smoke cigarettes?’ and for how many years. Participants who reported daily smoking were classified as addictive smokers.^15^ All other addictive behaviours were assessed using validated instruments: Pathological gambling was measured with the 2‐item Lie‐Bet Questionnaire; endorsement of at least one item indicated gambling behaviour.^28^ Alcohol use was screened using the 10‐item Alcohol Use Disorders Identification Test (AUDIT; score range 0–40); harmful/hazardous drinking was defined as ≥8 points, while dependence was defined as ≥13 in women and ≥15 in men.^29^ Drug‐related problems were assessed using the 11‐item Drug Use Disorder Identification Test (DUDIT; score range 0–44); scores of ≥6 (men) or ≥2 (women) indicated drug‐related problems, and ≥25 indicated dependence.^30^, ^31^ Eating disorders were screened using the 13‐item mYFAS 2.0 (score range 0–11); mild disorder was defined as a score of 2–3 with clinical significance and severe as 6–11 with significance.^32^ Internet addiction was assessed via the 20‐item Internet Addiction Test (IAT; score range 0–100), mild/moderate addiction was defined as scores of 31–79 and severe as 80–100.^33^, ^34^ Marital status was grouped into ‘in a relationship/married’ and ‘single/divorced/separated/widowed’. BMI was calculated from weight and height (kg/m^2^), and age and disease duration were derived from year of birth and diagnosis year to 2024. European regions followed the UN Geoscheme (Northern, Eastern, Southern, Western Europe).^35^ Thirty‐five participants with multiple skin conditions were excluded. DLQI scoring followed the official manual; missing values (<20%) were imputed using the mean of completed items per manual guidelines. Imputation was not possible for the single‐item happiness measure, which was analysed only in fully completed cases.

### Statistical analysis

Descriptive statistics were calculated for all variables (median [IQR], absolute and relative frequencies). Addiction prevalence across European regions and skin conditions was assessed using standard cut‐offs and reported in absolute and relative terms. Group differences were analysed via Kruskal–Wallis tests (continuous variables) and Pearson's chi‐squared tests (categorical variables). Univariate and multivariate logistic regressions identified sociodemographic and disease‐related factors associated with addiction, using conservative cut‐offs for alcohol, drug, internet addiction and eating disorders (e.g., mild eating disorder). Multicollinearity was evaluated via variance inflation factor (VIF <10 for continuous variables)^36^ and chi‐squared tests for categorical variables; no exclusions were necessary. Both unadjusted and adjusted odds ratios (OR, aOR) with 95% confidence intervals (CI) were reported. A significance level of α = 0.05 was used. Analyses were performed in SPSS (IBM Corp., Armonk, NY, USA). Regional boundaries were derived from European Commission Eurostat/GISCO data using QGIS v3.30.3 (QGIS Development Team).

## RESULTS

A total of 3953 patients from 20 European countries completed the survey; 368 were excluded (35 with multiple skin conditions, 304 with incomplete data and 29 without consent), resulting in 3585 patients for analysis. The median age was 43.0 years [30.0, 56.0], with 51.1% women. Most participants were employed (67.7%), lived in urban areas (71.7%) and were in a relationship or married (64.4%). The majority had psoriasis (44.7%), followed by AD (24.9%), HS (*n* = 383, 10.7%), urticaria (*n* = 373, 10.4%), vitiligo (*n* = 174, 4.9%) and AA (*n* = 159, 4.4%). Median disease duration was 14 years [4.0, 26.0] with a subjective severity score of 6.0 [4.0, 8.0] and DLQI of 6.0 [2.0, 12.0]. Geographically, 42.7% of patients were from Western, 21.7% from Eastern, 18.5% from Southern and 18.1% from Northern Europe. Demographic and disease characteristics varied by condition and regions, except for gender distribution (Tables 1 and 2).

**TABLE 1:** Characteristics of the participants in total as well as categorized in alopecia areata, atopic dermatitis, hidradenitis suppurativa, psoriasis, urticaria and vitiligo and differences between the different diseases.

	Total (*n* = 3,585)	Alopecia areata (*n* = 159)	Atopic dermatitis (*n* = 893)	Hidradenitis suppurativa (*n* = 383)	Psoriasis (*n* = 1,603)	Urticaria (*n* = 373)	Vitiligo (*n* = 174)	*p*‐Value
Age (years), median [IQR]	43.00 [30.00,56.00]	39.00 [26.00,51.00]	32.00 [24.00,50.00]	38.00 [29.00, 47.00]	49.00 [36.00, 61.00]	40.00 [27.50, 55.00]	43.00 [31.75, 56.25]	<0.001^a^
Gender, female *n* (%)	1,833 (51.1)	99 (62.3)	494 (55.3)	186 (48.6)	675 (42.1)	276 (74.0)	103 (59.2)	<0.001^b^
Body mass index, median [IQR]	26.20 [23.03,30.43]	24.22 [21.72, 27.76]	24.44 [21.63, 27.99]	28.65 [24.91, 33.98]	27.18 [24.11, 31.51]	25.31 [22.31, 29.08]	25.23 [22.42, 28.67]	<0.001^a^
Employment status, employed *n* (%)	2,426 (67.7)	123 (77.4)	587 (65.7)	288 (75.2)	1072 (66.9)	235 (63.0)	121 (69.5)	<0.001^b^
Residence, urban *n* (%)	2,570 (71.7)	122 (76.7)	655 (73.3)	267 (69.7)	1120 (69.9)	268 (71.8)	138 (79.3)	<0.001^b^
Marital status, in relationship/married *n* (%)	2,309 (64.4)	102 (64.2)	553 (61.9)	189 (49.3)	1125 (70.2)	240 (64.3)	100 (57.5)	<0.001^b^
European region, *n* (%)								<0.001^b^
South	664 (18.5)	33 (20.8)	154 (17.2)	51 (13.3)	328 (20.5)	61 (16.4)	37 (21.3)	
East	777 (21.7)	34 (21.4)	194 (21.7)	82 (21.4)	304 (19.0)	118 (31.6)	45 (25.9)	
West	1494 (41.7)	87 (54.7)	410 (45.9)	175 (45.7)	581 (36.2)	155 (41.6)	86 (49.4)	
North	650 (18.1)	5 (3.1)	135 (15.1)	75 (19.6)	390 (24.3)	39 (10.5)	6 (3.4)	
Disease duration, median [IQR]	14.00 [4.00, 26.00]	4.00 [2.00, 14.00]	22.00 [8.00, 32.00]	6.00 [3.00, 15.00]	18.00 [7.00, 29.00]	4.00 [2.00, 11.00]	10.50 [4.00, 24.00]	<0.001^a^
Subjective perceived disease severity, median [IQR]	6.00 [4.00, 8.00]	7.00 [4.00, 9.00]	6.00 [4.00, 8.00]	7.00 [4.00, 8.00]	6.00 [4.00, 8.00]	6.00 [4.00, 8.00]	5.00 [2.75, 6.25]	<0.001^a^
DLQI, median [IQR]	6.00 [2.00, 12.00]	5.00 [2.00, 11.00]	7.00 [3.00, 14.00]	10.00 [5.00, 17.00]	4.00 [1.00, 10.00]	6.00 [2.00, 12.00]	3.00 [1.00, 9.00]	<0.001^a^
Happiness, median [IQR]	7.00 [5.00, 8.00]	7.00 [5.00, 8.00]	7.00 [5.00, 8.00]	6.00 [4.00, 8.00]	7.00 [5.00, 8.00]	7.00 [5.00, 8.00]	7.00 [5.00, 8.00]	<0.001^a^

^a^ Kruskal–Wallis test.

^b^ Pearson χ^2^.

**TABLE 2:** Characteristics of the participants by the European regions.

	Southern Europe (*n* = 664)	Eastern Europe (*n* = 777)	Western Europe (*n* = 1494)	Northern Europe (*n* = 650)	*p*‐Value
Age (years), median [IQR]	42.00 [27.00, 56.00]	37.00 [25.00, 39.00]	44.00 [32.00, 58.00]	46.00 [33.00, 58.00]	<0.001^a^
Gender, female *n* (%)	333 (50.2)	396 (51.0)	761 (50.9)	343 (52.8)	0.805^b^
Body mass index, median [IQR]	25.40 [22.18, 29.73]	25.71 [22.31, 29.93]	26.22 [23.39, 30.72]	27.04 [24.09, 31.13]	<0.001^a^
Employment status, employed *n* (%)	407 (62.3)	490 (63.1)	1076 (72.0)	453 (69.7)	<0.001^b^
Residence, urban *n* (%)	541 (81.5)	599 (77.1)	932 (62.4)	498 (76.6)	<0.001^b^
Marital status, in relationship/married *n* (%)	443 (66.7)	494 (63.1)	926 (62.0)	446 (68.6)	0.014^b^
Chronic skin condition, *n* (%)					<0.001^b^
Alopecia areata	33 (5.0)	34 (4.4)	87 (5.8)	5 (0.8)	
Atopic dermatitis	154 (23.2)	194 (25.0)	419 (27.4)	135 (20.8)	
Hidradenitis suppurativa	51 (7.7)	82 (10.6)	175 (11.7)	75 (11.5)	
Psoriasis	28 (49.4)	304 (39.1)	581 (38.9)	390 (60.0)	
Urticaria	61 (9.2)	118 (15.2)	155 (10.4)	39 (6.9)	
Vitiligo	37 (5.6)	45 (5.8)	86 (5.8)	6 (0.9)	
Disease duration, median [IQR]	12.00 [4.00, 24.00]	11.00 [3.00, 23.00]	15.00 (5.00, 28.00)	18.00 [6.00, 33.00]	<0.001^a^
Subjective perceived disease severity, median [IQR]	6.00 [4.00, 8.00]	6.00 [3.00, 8.00]	6.00 [4.00, 8.00]	6.00 [4.00, 7.00]	<0.001^a^
DLQI, median [IQR]	5.00 [2.00, 10.00]	6.00 [2.00, 13.00]	6.00 [2.00, 13.00]	5.00 [1.00, 11.00]	<0.001^a^
Happiness, median [IQR]	7.00 [6.00, 8.00]	7.00 [5.00, 8.00]	7.00 [5.00, 8.00]	7.00 [5.00, 8.00]	<0.001^a^

^a^ Kruskal–Wallis test.

^b^ Pearson χ^2^.

### Addiction in skin conditions

**FIGURE 1:**
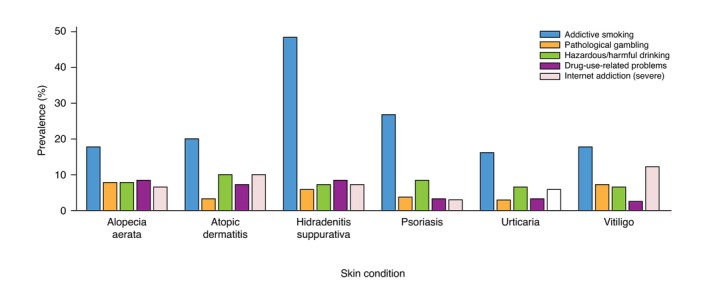
Comparison of the prevalence rates (%) of selected addictive behaviours—including addictive smoking, pathological gambling, hazardous/harmful drinking, drug‐use‐related problems and severe internet addiction—across six chronic skin diseases (Alopecia areata, Atopic dermatitis, Hidradenitis suppurativa, Psoriasis, Urticaria and Vitiligo).

Addictive smoking was reported by 25.7% of patients, ranging from 16.4% in urticaria to 48.6% in HS (*p* < 0.001, Figure 1 and Tables S3 and S4). Pathological gambling prevalence was 4.5%, highest in AA (8.2%) and vitiligo (7.5%) and lowest in AD (3.6%, *p* = 0.012). Hazardous/harmful drinking and alcohol dependence were reported by 8.8% and 2.5%, respectively, with no significant variation across conditions (*p* = 0.346). Drug‐use‐related problems and drug dependence were present in 5.3% and 0.4%, respectively, highest in AA (8.8%, 0.4%) and HS (8.6%, 0.5%) and lowest in vitiligo (2.9%, 0%, *p* < 0.001). Eating disorders were rare—mild (0.2%), moderate (0.3%), severe (1.3%)—with no significant differences across conditions (range: 0–2.9%, *p* = 0.105). Internet addiction was reported by 23.3% (mild/moderate) and 6.4% (severe), highest in AA (29.6%, 6.9%) and AD (28.8%, 10.3%) and lowest in PSO (19.9%, 3.3%, *p* < 0.001).

### Addiction in EU regions

**FIGURE 2:**
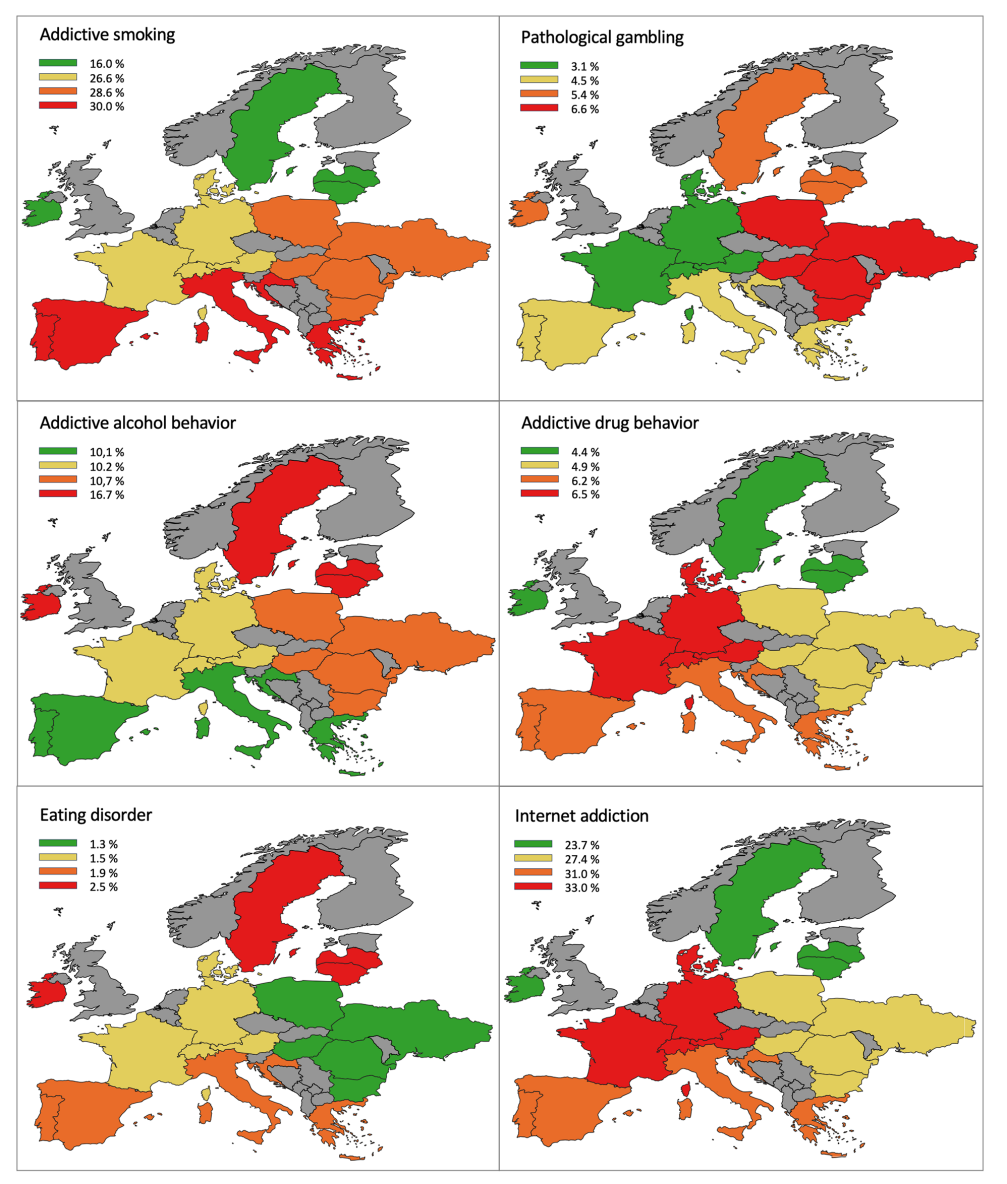
Visualization of the prevalence of addictive smoking, pathological gambling, addictive alcohol behaviour, addictive drug behaviour, eating disorder and internet addiction in Southern, Eastern, Western and Northern Europe. Percentages of *n* = 3585. Addictive alcohol behaviour contains hazardous/harmful drinking and alcohol dependence. Addictive drug behaviour contains drug‐use‐related problems and drug dependence. Eating disorder contains mild, moderate and severe eating disorders. Internet addiction contains mild/moderate and severe addiction. Colours are only interpretable within one addiction. Grey = not assessed.

Addictive smoking prevalence ranged from 16.0% to 30.0%, highest in Southern Europe (*p* < 0.001, Figure 2 and Table 3). Pathological gambling varied significantly (*p* < 0.001), peaking in Eastern Europe (6.6%) and lowest in Western Europe (3.1%). Hazardous/harmful drinking was most common in Northern Europe (14.3%), while alcohol dependence peakedin Western Europe (3.2%). Drug‐use‐related problems showed minor variation, highest in Western Europe (6.0% for problems and 0.5% for dependence) and lowest in Northern Europe (4.2% and 0.2%, *p* = 0.379). Eating disorders showed minimal regional differences (0.1–2.0%, *p* = 0.649). Internet addiction varied regionally, with mild/moderate and severe forms most prevalent in Western and Southern Europe (26.2%/23.2%)and (0.6–8%/7.8%, respectively, *p* < 0.001).

**TABLE 3:** Prevalence of addictive smoking, pathological gambling, hazardous/harmful drinking, alcohol dependence, drug‐use‐related problems, drug dependence, eating disorders and internet addiction by European region.

	Southern Europe (*n* = 664)	Eastern Europe (*n* = 777)	Western Europe (*n* = 1494)	Northern Europe (*n* = 650)	*p*‐Value^a^
Addictive smoking, *n* (%)	199 (30.0)	222 (28.6)	397 (26.6)	104 (16.0)	<0.001
Pathological gambling, *n* (%)	30 (4.5)	51 (6.6)	46 (3.1)	35 (5.4)	<0.001
Addictive alcohol behaviour, *n* (%)					<0.001
Hazardous/harmful drinking	48 (7.2)	69 (8.9)	105 (7.0)	93 (14.3)	
Alcohol dependence	13 (2.9)	14 (1.8)	48 (3.2)	15 (2.3)	
Addictive drug behaviour, *n* (%)					0.379
Drug‐use‐related problem	38 (5.7)	36 (4.6)	90 (6.0)	27 (4.2)	
Drug dependence	3 (0.5)	2 (0.3)	8 (0.5)	1 (0.2)	
Eating disorder, *n* (%)					0.649
Mild	2 (0.3)	2 (0.3)	2 (0.1)	1 (0.2)	
Moderate	1 (0.2)	1 (0.1)	6 (0.4)	2 (0.3)	
Severe	9 (1.4)	7 (0.9)	16 (1.1)	13 (2.0)	
Internet addiction, *n* (%)					<0.001
Mild	154 (23.2)	165 (21.2)	392 (26.2)	125 (19.2)	
Severe	52 (7.8)	48 (6.2)	101 (6.8)	29 (4.5)	

^a^ Pearson χ^2^.

### Influential factors for addictions in patients with chronic skin conditions

There were no substantial differences between the univariate analyses (Table S2) and the multivariate regression analysis (Tables S3 and S4).

*Addictive Smoking* Higher odds of addictive smoking were found among males, individuals in rural areas, those not in a relationship and patients with higher subjective severity and DLQI scores (aOR range: 1.023–1.417, *p* = 0.001–0.058). HS (aOR: 3.285 [2.474–4.361], *p* < 0.001) and PSO (1.630 [1.314–2.024], *p* < 0.001) were also associated with increased risk compared to AD. Conversely, unemployment, longer disease duration and residence in Western or Northern Europe (vs. Eastern) were linked to lower odds (aOR range: 0.392–0.991, *p* = 0.001–0.034). Age, BMI and happiness showed no significant association. Adjusted *R* ^2^ = 0.12.

*Pathological gambling* Male gender and specifically AA, HS and vitiligo, compared to AD, were associated with higher odds of pathological gambling (1.888 [1.041, 3.424] ≤ aOR ≤ 3.468 [1.700, 7.407], 0.001 ≤ *p* ≤ 0.036). Higher age (0.985 [0.972, 0.997], *p* = 0.018) and not being in a relationship or marriage (1.402 [0.996, 1.973], *p* = 0.053) were associated with a lower likelihood of pathological gambling. BMI, employment status, residence, disease severity and happiness showed no impact on pathological gambling. Adjusted *R* ^2^ was 0.12.

*Addictive alcohol behaviour* Males, individuals not in a relationship, and individuals from Northern Europe compared to Southern were more likely to exhibit addictive behaviour (1.500 [1.197, 1.882] ≤ aOR ≤ 3.027 [2.394, 3.828], *p* < 0.001). Higher BMI (0.966 [0.946, 0.986], *p* < 0.001) and unemployment (0.731 [0.568, 0.941], *p* = 0.015) were associated with no addictive alcohol behaviour. Among skin conditions, only HS showed a lower risk than AD (0.661 [0.438, 0.997], *p* = 0.048). Higher happiness was associated with lower addictive consumption (0.938 [0.891, 0.987], *p* = 0.014). Adjusted *R* ^2^ was 0.11.

*Addictive drug behaviour* Older age, higher BMI, living in rural areas and being happier were associated with lower odds of addictive drug behaviour (0.655 [0.453, 0.947] ≤ aOR ≤ 0.949 [0.936, 0.962], 0.001 ≤ *p* ≤ 0.040). Being affected by HS compared to AD (1.625 [0.987, 2.674], *p* = 0.053) and higher DLQI scores (1.049 [1.024, 1.074], *p* < 0.001) were associated with addictive drug behaviour. Regionally, individuals from Eastern Europe, compared to Southern, showed a tendency for higher risk for addictive drug use (0.625 [0.390, 1.002], *p* = 0.051). Adjusted *R* ^2^ was 0.14.

*Eating disorders* Older age, male gender and being happier were associations of decreased eating disorder disease (0.416 [0.232, 0.744] ≤ aOR ≤ 0.957 [0.937, 0.978], 0.001 ≤ *p* ≤ 0.003). Unemployment, living in a rural area and being affected by vitiligo compared to AD were associated with a higher risk (1.101 [1.071, 1.132] ≤ aOR ≤ 5.367 [1.858, 15.502], 0.001 ≤ *p* ≤ 0.002). Adjusted *R* ^2^ was 0.18.

*Internet addiction* Older age, rural residence and higher happiness were associated with lower odds of internet addiction (aOR range: 0.718–0.947, *p* < 0.001), whereas male gender, being single and higher DLQI were associated with increased risk (aOR range: 1.016–1.299, *p* = 0.002–0.014). Compared to AD, HS was linked to lower odds (aOR: 0.677 [0.504–0.911], *p* = 0.010), whereas vitiligo was linked to higher odds (1.477 [1.012–2.156], *p* = 0.043). Regionally, risk was lower in Eastern Europe (0.640 [0.498–0.821], *p* < 0.001) and higher in Western Europe (1.266 [1.014–1.582], *p* = 0.037) compared to Southern. Adjusted *R* ^2^ = 0.21.

## DISCUSSION

The findings from this pan‐European cross‐sectional study provide novel insights into the comorbidity of addictive behaviours and chronic skin diseases, demonstrating that various forms of addiction are present among patients with dermatological conditions. The study revealed notably high prevalence rates of smoking, gambling, alcohol use, drug dependence and internet addiction, particularly among patients with PSO, HS and AD, which emphasizes the critical need for new approaches to dermatological care that account for addictive behaviour.

The overall smoking prevalence (25.7%) in this cohort exceeds the 19% reported in the general European population.^37^ This is particularly relevant for patients with HS and PSO, where smoking exacerbates disease activity and may reduce treatment efficacy.^38^, ^39^ Smoking's role in promoting inflammation in skin diseases is well established,^9^, ^40^, ^41^ and its association with higher DLQI scores in this study underscores its negative impact. These findings align with previous studies showing better treatment outcomes in non‐smokers.^39^, ^42^ The elevated smoking rates in Southern Europe highlight the need for targeted cessation programs, especially in culturally entrenched regions. In contrast, the lower prevalence in Northern Europe may reflect stricter tobacco regulations, while other regions showed smoking rates exceeding those of the general EU population.^37^

The study identifies several sociodemographic and clinical factors associated with addiction risk in patients with chronic skin conditions. Age showed an inverse relationship, with older individuals less likely to engage in addictive behaviours – possibly reflecting generational differences in substance use and coping.^43^ However, the higher prevalence of internet addiction among younger cohorts highlights the need for age‐specific prevention strategies. Sex also played a role: men were more prone to smoking, gambling and alcohol use, consistent with general population trends,^15^ whereas women showed a higher risk for eating disorders, aligning with broader epidemiological data.^44^ Though eating disorder prevalence was low in this cohort, potential underreporting—especially in sensitive populations—should be considered.^45^ Unexpectedly, unemployment was linked to lower addiction risk, except in eating disorders, where non‐employed individuals showed higher prevalence. This may reflect complex psychosocial dynamics, such as greater work‐related stress among employed individuals^46^ although the role of employment in addiction risk remains debated.^47^ Finally, being single was associated with higher addiction risk, possibly due to increased isolation and emotional distress.^48^ Disease duration showed only a slight increase in addiction risk, while subjective severity had no clear impact. In contrast, DLQI scores—reflecting reduced quality of life—were strongly associated with smoking, drug use and internet addiction, suggesting addiction may serve as a maladaptive coping response.^3,^ ^49^ Variability in addiction risk across skin conditions may be partly explained by differing personality traits, such as neuroticism or stress sensitivity, which influence coping styles and addiction.^50^, ^51^

Higher happiness scores were associated with lower addiction risk, suggesting a potential protective effect. Whether happiness buffers against addiction or results from its absence remains unclear, but the link highlights the importance of holistic, person‐centred dermatological care that includes mental health support.^52^, ^53^ Regional differences in addiction prevalence highlight the influence of cultural, socio‐economic and policy‐related factors. For example, elevated rates of smoking and pathological gambling in Eastern Europe may reflect region‐specific economic stressors or greater access to gambling.^54^ These findings emphasize the need to tailor prevention and treatment strategies to the distinct social and economic contexts of each European region.

Several limitations should be considered when interpreting our findings. First, this was not a population‐based study, and the sample may not represent the general population with skin diseases. Recruitment was conducted exclusively in tertiary dermatology centres, which may bias the sample toward patients with more severe or complex conditions. Second, the absence of a control group prevents direct comparison with individuals without skin diseases and limits causal inference. At the same time, selection bias is possible, as individuals with addictions may have avoided participation or underreported behaviors despite anonymity. Third, the proportion of missing data was not recorded systematically, and the response rate could not be determined, which may introduce reporting bias. Fourth, although descriptive differences between regions were observed, the study design does not allow for definitive conclusions on regional variations. Future research needs to employ population‐based designs with standardized sampling and include appropriate control groups to validate and expand on these findings.

The relatively small *R* ^2^ values in the regression models indicate that unmeasured factors—such as stigma, loneliness and hopelessness—may also contribute to the high addiction rates. Patients with visible skin conditions like AD and vitiligo often face social stigma, exacerbating psychological distress and potentially promoting addictive behaviours.^55^, ^56^, ^57^, ^58^ Future research should focus on these psychosocial dimensions, particularly the roles of stigma and social support in addiction risk. Despite these limitations, this large, multi‐centre study offers valuable insights into the complex relationship between skin diseases and addiction across Europe. Its comprehensive assessment of multiple addictive behaviours highlights the importance of integrating addiction screening and management into dermatological care—especially for patients with high DLQI scores or those in regions with elevated rates of smoking and gambling.

## CONCLUSIONS

In this multicentre tertiary care study across 20 European countries, addictive disorders were common among patients with chronic skin diseases. The observed associations underline the relevance of routine screening for addictive behaviours in dermatological settings and the potential value of multidisciplinary care. However, the absence of a control group, the lack of population‐based sampling and recruitment bias towards more severe cases necessitate cautious interpretation. Future studies using population‐based approaches and appropriate comparison groups are essential to confirm these patterns and inform targeted interventions.

## AUTHOR CONTRIBUTIONS

SZ and AZ contributed to the study and the design. MS, AS, RM, EM, EJ, GS, FL, CK, AS, CG, BK, EP, AZJ, KT, GC, MJB, SF, ACP, FS, MT, WZ, KG, AS, GD, AC, IH, IM, AV, LP, JS, BJ, PS, SLH, SSB, CP, JJ, AW, LS, JJ, ET, LC, GLF, PC, LK, JW, KT, JTM, LVMD, DM, SB, COC, RD, ST, CV, ELPP, MPK, OSE, KH, NY, HACL, ASB, AS, TT, ZB, ASV, LM, NM and ES contributed to the data acquisition; SZ and AZ contributed to the analysis and data interpretation. SZ and AZ drafted the manuscript. MS, AS, RM, EM, EJ, GS, FL, CK, AS, CG, BK, EP, AZJ, KT, GC, MJB, SF, ACP, FS, MT, WZ, KG, AS, GD, AC, IH, IM, AV, LP, JS, BJ, PS, SLH, SSB, CP, JJ, AW, LS, JJ, ET, LC, GLF, PC, LK, JW, KT, JTM, LVMD, DM, SB, COC, RD, ST, CV, ELPP, MPK, OSE, KH, NY, HACL, ASB, AS, TT, ZB, ASV, LM, NM and ES provided critical feedback and helped shape the research, analysis and manuscript. All authors approved the submitted version.

## FUNDING INFORMATION

This study was funded by the EADV (PPRC‐2022‐19).

## CONFLICT OF INTEREST STATEMENT

SZ, MS, AS, RM, CK, AS, CG, AZJ, KT, GC, PL, WZ, KG, GD, AV, LP, BJ, SSB, AW, LS, MJB, SF, AS, ET, GLF, PC, LK, DM, COC, RD, ELPP, HACL, AS, TT, ASV, LM and ES have no conflict of interest to declare. CH has been a speaker for Janssen and/or has received support for attending meetings and/or travel from Almirall, Janssen, LEO Pharma. EM has received speaker honoraria and/or been a consultant and/or support for attending meetings and/or participated on a data safety monitoring from Amgen, Biolane, Almirall, Sanofi, Leo Pharma, Novartis, Janssen Cilag, UCB. EJ has received speaker honoraria and/or been a consultant and/or been co‐investigator in clinical trials for AbbVie, ACO, Almirall, Amgen, the Swedish Asthma and Allergy Foundation, Galenica, LEO Pharma, Lakartidningen, Novartis, Pfizer, Sanofi‐Genzyme and the Swedish Society for Dermatology and Venereology. JL has been a speaker and/or consultant for AbbVie, Janssen Cilag/Johnson & Johnson, Sanofi, Leo Pharma, Galderma, Lilly and UCB Pharma. GS has received speaker honoraria and/or been in advisory boards for AbbVie, Eli Lilly, LEO Pharma and Almirall. FL has received payments for consulting and participation in advisory boards and/or honoraria for presentations/lectures and/or support for attending meetings/travel from Almirall, Celgene, Eli Lilly, Galderma, Menlo Therapeutics, Novartis, Pelpharma, Pfizer, Sanofi, Trevi Therapeutics, Vifor Pharma, Amgen, AbbVie, Janssen‐Cilag and LEO Pharma. BK has received research support from/was a principal investigator (clinical trials) for AbbVie, Almirall, Janssen, Merck Sharpe Dohme, MoonLake Immunotherapeutics, Novartis, Pfizer and UCB; been a consultant for AbbVie, Almirall, Celgene, Janssen, Leo, Lilly, MC2 therapeutics, Merck Sharpe Dohme, MoonLake Immunotherapeutics, Novartis, Pfizer and UCB; received honoraria from AbbVie, Almirall, Celgene, Janssen, Leo, Lilly, MC2 therapeutics, MoonLake Immunotherapeutics, Novartis, Pfizer, Union and UCB and been on scientific advisory boards for AbbVie, Almirall, Celgene, GSK, Janssen, Lilly, MoonLake Immunotherapeutics, Novartis, Pfizer and UCB. EP has been an advisor for UCB Pharma and received honoraria from Janssen and UCB Pharma, and participated in clinical trials for AbbVie, MoonLake and UCB Pharma. ACP has served as an advisor and/or received honoraria as a speaker and/or received travel grants and/or participated in clinical trials from the following companies: AbbVie, ALK‐Abelló, Bristol Myers Squibb, Boehringer Ingelheim, InfectoPharm, LEO Pharma, Novartis and UCB. FS has received speaker's honoraria and/or support for attending meetings/travel from Astra Zeneca and/or is a member of the local ethical committee Freiburg. MT has received speaker's honoraria and/or support for attending meetings/travel from AbbVie, Bausch Health, Bio derma, Eli Lilly, Fabre, la Roche‐Posay, Pfizer, LEO Pharma, Mead Johnson, Sanofi and/or has been a board member of Sanofi, Bausch Health, AbbVie, LEO Pharma and/or has been chair of ETFAD, board member of ISAD, secretary of PSD. SLH is PI for atopic dermatitis Clinical Study (AbbVie, Amgen, Nektar) and was PI for chronic spontaneous urticaria Clinical study (Novartis) and lecturer for Novartis, AbbVie, Sanofi, Pliva, Bayer and Berlin‐Chemie. AC has been an advisor, speaker and/or consultant for AbbVie, Almirall, Amgen, Boehringer‐Ingelheim, Eli Lilly, Janssen‐Cilag, LEO Pharma, Novartis, Sanofi, UCB. IH has been a consultant and/or a speaker and/or has received support for attending meetings/travels and/or has been a board member of the following companies: Janssen, AbbVie, Novartis, Stada. IM has received honoraria for lectures/educational events and has been supported for attending meetings/travels from Johnson&Johnson, AbbVie, Novartis, Stada. JCS has been a consultant and advisory board member of AbbVie, LEO Pharma, Novartis, Pierre Fabre, Sanofi‐Genzyme, UCB, Sandoz, Almirall, Boehringer‐Ingelheim and Galderma; speaker for AbbVie, Almirall, Boehringer‐Ingelheim, Janssen, Eli Lilly, LEO Pharma, Novartis, Pfizer, Pierre Fabre, Sanofi‐Genzyme and UCB; investigator for AbbVie, Aceleryn, Almirall Hermal, Amgen, AnaptysBio, Argenx, Aslan, Boehringer Ingelheim, Biocom, Bio Thera, Bristol Myers Squibb, Celtrion, CuraTeQ Biologics, DICE Therapeutics, Eli Lilly, Helm AG, Galapagos, Galderma, Janssen, Incyte, InflaRX, Kliniksa, Kymab Limited, LEO Pharma, Medimmune, Menlo Therapeutics, Merck, Moonlake, Novartis, Pierre Fabre, Pfizer, Regeneron Pharmaceuticals, Takeda, Teva, Trevi Therapeutics, UCB Biopharm, Uni Therapeutics and Ventyx Bioscience. JJ has received honoraria for presentations/speaking from AbbVie and Almirall and/or has received support for attending meetings and/or travel from Lilly and Amgen. CP has received grants and/or speaker's honoraria and/or support for attending meetings and/or travel from the following companies: Almirall, BMS, MSD, Pierre Fabre, Novartis, AbbVie, Sanofi, Galderma, Leo Pharma, Eli Lilly, Pfizer, DSD, Incyte, Takeda, Astra Zeneca, Pelpharma, Amazentis, Janssen, Merck, Stada. LC has been a speaker for AbbVie and Almirall. KT has received support for attending meetings and/or travel and/or participated in clinical trials of the following companies: Sanofi‐Aventis, German Dermatological Society, Boehringer Ingelheim, Lilly, Novartis, Janssen‐Cilag, Aristea Therapeutics, LEO Pharma, Almirall, InfectoPharm, Celldex, Allakos, Bristol‐Myers. JTM has served as advisor and/or received speaking fees and/or participated in clinical trials sponsored by AbbVie, Almirall, Amgen, BMS, Celgene, Eli Lilly, Incyte, Janssen‐Cilag, LEO Pharma, MSD, Novartis, Pfizer, Pierre Fabre, Roche, Sanofi, UCB. JW received speaker's honoraria and/or travel support from AbbVie, Almirall, BMS, Boehringer Ingelheim, Janssen, Infectopharm, Leo, Lilly, Mibe, MSD, Novartis, Pfizer and Sanofi. LVM has served as advisor and/or received speaking fees and/or participated in clinical trials sponsored by Almirall, Amgen, Bristol‐Myers Squibb, Eli Lilly, Incyte, MSD, Novartis, Pierre Fabre, Roche and Sanofi. MPK has received travel support and honoraria from AbbVie, Janssen, Eli Lilly, LEO Pharma and Novartis and has participated on Advisory Boards for UCB. KH has served as an advisor and/or paid speaker for and/or participated in clinical trials sponsored by Almirall‐Hermal, Amgen, LEO Pharma, Celgene, UCB, Sanofi. With no relation to the present manuscript, NY has served as an advisor and/or received speaking fees and/or participated in clinical trials sponsored by AbbVie, Almirall, Amgen, Boehringer Ingelheim, Bristol‐Myers Squibb, Celgene, Eli Lilly, Galderma, LEO Pharma, Incyte, Janssen‐Cilag, MSD, Novartis, Pfizer, Sanofi and UCB. SB has received research grants from Amgen. ST has received research grants from Sanofi and Novartis Foundation and/or served as consultant and lecturer for Lilly Pharma, Leo Pharma, Janssen and Sanofi. CV is president of Nordic Dermatology Associations and has received honoraria for lectures/presentations and/or support for attending meetings/travel from Pfizer, AbbVie, Amirall, Sanofi, LEO Pharma, Chiesi, Galderma. AS has been an advisor and/or received speaker's honoraria and/or received travel grants from the following companies: AbbVie, Janssen Cilag, Pfizer. ZB has been a speaker for Novartis, AstraZeneca, Glaxo and Berlin‐Chemie Menarini. NM has been an advisor, speaker and/or consultant for AbbVie, Almirall, Amgen, Boehringer Ingelheim, Bristol Myers Squibb, Celgene, Janssen, La Roche‐Posay, LEO Pharma, Lilly, Novartis, Pfizer, Sanofi, Dr. Wolff Group and UCB Pharma. TB has received grants and/or consulting fees and/or speaker's honoraria from the following companies: Almirall, Celgene‐BMS, Lilly, Novartis, Sanofi‐Genzyme, Regeneron, Viatris, AbbVie, Alk‐Abello, Boehringer‐Ingelheim, LEO Pharma, GSK, Galderma. Furthermore, TB was president of the German Society of Dermatology (unpaid) and/or is a board member of Alk‐Abello, Almirall, Boehringer‐Ingelheim, Leo Pharma, Lilly, Novartis, Sanofi‐Genzyme, Viatris. AZ has been an advisor and/or received speaker's honoraria and/or received grants and/or participated in clinical trials of the following companies: AbbVie, Almirall, Amgen, Beiersdorf Dermo Medical, Bencard Allergie, BMS, Celgene, Eli Lilly, GSK, Janssen Cilag, Leo Pharma, Miltenyi Biotec, Novartis, Pfizer, Sanofi‐Aventis, Takeda Pharma, UCB Pharma.

## ETHICAL APPROVAL

Ethical adherence was ensured, including the lead ethical committee at the Medical Faculty at the Technical University of Munich, Germany (reference 2023‐308‐S‐KH).

## ETHICS STATEMENT

Not applicable.

## Supporting information


Table S1.

## Data Availability

The data that support the findings of this study are available from the corresponding author upon reasonable request.
