# Supplementary material for: Addiction and chronic skin diseases: A Pan‐European study on prevalence, associations and patient impact
Source: J Eur Acad Dermatol Venereol. 2025 Dec 14;40(8):1392–405. doi: 10.1111/jdv.70245 (PMC13425250; doi:10.1111/jdv.70245)
Supplement: Supplementary file 1 — Table S1. [file JDV-40-1392-s001.docx]

**Supplementary material**

Table S1 Overview of the countries, number of centers, number of participants, and European regions

|  | Country | Number of centers | Number of patients | European region |
| --- | --- | --- | --- | --- |
| 1 | Austria | 3 | 305 | Western |
| 2 | Bulgaria | 1 | 54 | Eastern |
| 3 | Croatia | 2 | 118 | Southern |
| 4 | Denmark | 2 | 73 | Western |
| 5 | France | 1 | 159 | Western |
| 6 | Germany | 8 | 841 | Western |
| 7 | Greece | 1 | 50 | Southern |
| 8 | Hungary | 2 | 150 | Eastern |
| 9 | Ireland | 2 | 172 | Northern |
| 10 | Italy | 4 | 295 | Southern |
| 11 | Latavia | 1 | 99 | Northern |
| 12 | Lithuania | 1 | 99 | Northern |
| 13 | Malta | 1 | 105 | Southern |
| 14 | Poland | 4 | 398 | Eastern |
| 15 | Portugal | 1 | 45 | Southern |
| 16 | Romania | 1 | 89 | Eastern |
| 17 | Spain | 1 | 50 | Southern |
| 18 | Sweden | 2 | 280 | Northern |
| 19 | Switzerland | 2 | 118 | Western |
| 20 | Ukraine | 1 | 85 | Eastern |

Table S2 Results of the univariate regression with the different addictions as the dependent variable and sociodemographic and disease-related characteristics as independent variables.

|  | Addictive Smoking OR [95%-CI] | Pathological gambling OR [95%-CI] | Addictive alcohol behavior OR [95%-CI] | Addictive drug behavior OR [95%-CI] | Eating disorder  OR [95%-CI] | Internet addiction  OR [95%-CI] |
| --- | --- | --- | --- | --- | --- | --- |
| **Age** (years) | 0.995 [0.990, 1.000] | 0.987 [0.977, 0.997] | 0.994 [0.988, 1.000] | 0.948 [0.938, 0.959] | 0.981 [0.965, 0.997] | 0.949 [0.944, 0.954] |
| **Gender** (ref. female) | 1.551 [1.300, 1.757] | 2.670 [1.889, 3.773] | 2.737 [2.188, 3.424] | 0.917 [0.691, 1.216] | 0.422 [0.242, 0.733] | 1.061 [0.919, 1.224] |
| **Body mass index** | 1.008 [0.997, 1.020] | 0.998 [0.974, 1.024] | 0.976 [0.959, 0.994] | 0.931 [0.905, 0.958] | 1.093 [1.064, 1.122] | 0.966 [0.954, 0.978] |
| **Employment** **status** (ref. employed) | 0.815 [0.692, 0.960] | 0.932 [0.662, 1.310] | 0.706 [0.559, 0.892] | 0.796 [0.582, 1.090] | 1.629 [0.981, 2.705] | 0.859 [0.736, 1.003] |
| **Residence**, (ref. urban) | 1.227 [1.042, 1.444] | 0.823 [0.572, 1.186] | 0.753 [0.592, 0.959] | 0.598 [0.420, 0.851] | 0.603 [0.320, 1.137] | 0.702 [0.595, 0.829] |
| **Marital** **status**, (ref. in relationship/married) | 1.426 [1.223, 1.663] | 1.514 [1.102, 2.079] | 1.470 [1.192, 1.813] | 1.861 [1.402, 2.469] | 1.954 [1.182, 3.231] | 1.763 [1.522, 2.042] |
| **Chronic skin condition** (ref. atopic dermatitis) *Alopecia areata Hidradenitis suppurativa Psoriasis Urticaria Vitiligo* | 0.884 [0.572, 1.364] 3.740 [2.888, 4.844] 1.475 [1.211, 1.797] 0.774 [0.563, 1.066] 0.859 [0.563, 1.309] | 2.396 [1.228, 4.673] 1.799 [1.045, 3.097] 1.192 [0.777, 1.829] 0.894 [0.456, 1.756] 2.173 [1.116, 4.230] | 0.757 [0.442, 1.296] 0.737 [0.504, 1.077] 0.804 [0.629, 1.028] 0.552 [0.363, 0.841] 0.596 [0.340, 1.047] | 1.244 [0.692, 2.235] 1.201 [0.785, 1.838] 0.505 [0.356, 0.716] 0.535 [0.306, 0.935] 0.353 [0.140, 0.889] | * 1.367 [0.534, 3.499] 1.305 [0.660, 2.580] 1.609 [0.652, 3.969] 3.077 [1.194, 7.931] | 0.895 [0.631, 1.270] 0.669 [0.518, 0.864] 0.471 [0.394, 0.563] 0.611 [0.470, 0.794] 0.976 [0.699, 1.262] |
| **Disease** **duration** (years) | 0.988 [0.983, 0.993] | 0.999 [0.988, 1.009] | 1.003 [0.996, 1.009] | 0.987 [0.978, 0.997] | 1.000 [0.984, 1.016] | 0.985 [0.980, 0.990] |
| **Subjective** **severity** | 1.071 [1.040, 1.104] | 0.980 [0.922, 1.041] | 1.004 [0.965, 1.045] | 0.955 [0.905, 1.008] | 1.058 [0.957, 1.169] | 1.008 [0.980, 1.036] |
| **DLQI** | 1.036 [1.026, 1.046] | 1.013 [0.992, 1.034] | 1.010 [0.996, 1.024] | 1.042 [1.024, 1.060] | 1.049 [1.017, 1.081] | 1.025 [1.016, 1.035] |
| **Happiness** | 0.933 [0.904, 0.964] | 0.959 [0.896, 1.027] | 0.943 [0.902, 0.985] | 0.910 [0.858, 0.966] | 0.800 [0.724, 0.885] | 0.929 [0.900. 0.959] |
| **European region** (ref. Southern)  *Eastern Western Northern* | 0.935 [0.744, 1.174] 0.846 [0.691, 1.035] 0.445 [0.341, 0.582] | 1.485 [0.934, 2.360] 0.671 [0.430, 1.073] 1.203 [0.729, 1.983] | 1.182 [0.835, 1.674] 1.128 [0.826, 1.541] 1.970 [1.410, 2.753] | 0.781 [0.496, 1.230] 1.067 [0.732, 1.554] 0.684 [0.418, 1.120] | 0.708 [0.304, 1.650] 0.887 [0.441, 1.785] 1.371 [0.644, 2.921] | 0.840 [0.669, 1.054] 1.095 [0.900, 1.333] 0.690 [0.541, 0.881] |

*eating disorders were not observed in patients with alopecia areata; OR, odds ratio; CI, confidence interval

Table S3 Prevalence of addictive smoking, pathological gambling, hazardous/harmful drinking, alcohol dependence, drug-use-related problems, drug dependence, eating disorders, and internet addiction: overall and by chronic skin condition

|  | Total | Alopecia areata | Atopic dermatitis | Hidradenitis suppurativa | Psoriasis | Urticaria | Vitiligo | p-value^a^ |
| --- | --- | --- | --- | --- | --- | --- | --- | --- |
| n | 3,585 | 159 | 893 | 383 | 1603 | 373 | 174 |  |
| **Addictive smoking**, n (%) | 922 (25.7) | 29 (18.2) | 180 (20.2) | 186 (48.6) | 435 (27.1) | 61 (16.4) | 31 (17.8) | <.001 |
| **Pathological gambling**, n (%) | 162 (4.5) | 13 (8.2) | 32 (3.6) | 24 (6.3) | 68 (4.2) | 12 (3.2) | 13 (7.5) | .012 |
| **Addictive alcohol behavior**, n (%)  Hazardous/harmful drinking  Alcohol dependence | 315 (8.8)  90 (2.5) | 13 (8.2)  4 (2.5) | 94 (10.5)  28 (3.1) | 30 (7.8)  10 (2.6) | 141 (8.8)  40 (2.5) | 25 (6.7)  5 (1.3) | 12 (6.9)  3 (1.7) | .346 |
| **Addictive drug behavior**, n (%)  Drug-use-related problem  Drug dependence | 191 (5.3)  14 (0.4) | 14 (8.8)  1 (0.6) | 67 (7.5)  2 (0.2) | 33 (8.6)  2 (0.5) | 59 (3.7)  6 (0.4) | 13 (3.5)  3 (0.8) | 5 (2.9)  0 (0) | <.001 |
| **Eating disorder**, n (%)  Mild  Moderate  Severe | 7 (0.2)  10 (0.3)  45 (1.3) | 0 (0)  0 (0)  0 (0) | 3 (0.3)  1 (0.1)  8 (0.9) | 0 (0)  2 (0.5)  5 (1.3) | 4 (0.2)  2 (0.1)  22 (1.4) | 0 (0)  3 (0.8)  5 (1.3) | 0 (0)  2 (1.1)  5 (2.9) | .105 |
| **Internet addiction**, n (%)  Mild  Severe | 836 (23.3)  230 (6.4) | 47 (29.6)  11 (6.9) | 257 (28.8)  92 (10.3) | 87 (22.7)  28 (7.3) | 319 (19.9)  53 (3.3) | 81 (21.7)  24 (6.4) | 45 (25.9)  22 (12.6) | <.001 |

^a^*Pearson Chi^2^*

Table S4 Results of the multiple regression with the different addictions as the dependent variable and sociodemographic and disease-related characteristics as independent variables.

|  | Addictive smoking  aOR [95%-CI] | Pathological gambling  aOR [95%-CI] | Addictive alcohol behavior  aOR [95%-CI] | Addictive drug behavior  aOR [95%-CI] | Eating disorder  aOR [95%-CI] | Internet addiction  aOR [95%-CI] |
| --- | --- | --- | --- | --- | --- | --- |
| **Age** (years) | 1.000 [0.995, 1.004] | 0.985 [0.972, 0.997] | 0.998 [0.991, 1.006] | 0.949 [0.936, 0.962] | 0.957 [0.937, 0.978] | 0.947 [0.941, 0.953] |
| **Gender** (ref. female) | 1.417 [1.205, 1.666] | 2.946 [2.056,4.220] | 3.027 [2.394, 3.828] | 1.072 [0.792, 1.451] | 0.416 [0.232, 0.744] | 1.287 [1.095, 1.514] |
| **Body mass index** | 0.991 [0.978, 1.004] | 0.997 [0.969, 1.025] | 0.966 [0.946, 0.986] | 0.949 [0.932, 0.986] | 1.101 [1.071, 1.132] | 0.996 [0.983, 1.009] |
| **Employment** **status** (ref. employed) | 0.825 [0.691, 0.986] | 1.071 [0.744, 1.542] | 0.731 [0.568, 0.941] | 0.914 [0.650, 1.286] | 2.004 [1.147, 3.501] | 0.879 [0.731, 1.022] |
| **Residence**, (ref. urban) | 1.251 [1.051, 1.490] | 0.945 [0.649, 1.377] | 0.793 [0.616, 1.022] | 0.655 [0.453, 0.947] | 0.547 [0.281, 1.066] | 0.718 [0.599, 0.861] |
| **Marital** **status**, (ref. in relationship/married) | 1.360 [1.152, 1.606] | 1.402 [0.996, 1.973] | 1.500 [1.197, 1.882] | 1.266 [0.929, 1.724] | 1.462 [0.849, 2.516] | 1.299 [1.101, 1.533] |
| **Chronic skin condition** (ref. atopic dermatitis) *Alopecia areata Hidradenitis suppurativa Psoriasis Urticaria Vitiligo* | 0.760 [0.485, 1.192] 3.285 [2.474, 4.361] 1.630 [1.314, 2.024] 0.728 [0.518, 1.023] 0.850 [0.551, 1.313] | 3.468 [1.700, 7.074] 1.888 [1.041, 3.424] 1.311 [0.819, 2.099] 1.401 [0.678, 2.895] 3.079 [1.510, 6.277] | 0.865 [0.491, 1.521] 0.661 [0.438, 0.997] 0.779 [0.592, 1.025] 0.744 [0.472, 1.172] 0.687 [0.382, 1.237] | 1.662 [0.874, 3.159] 1.625 [0.987, 2.674] 1.146 [0.763, 1.720] 0.852 [0.461, 1.575] 0.519 [0.199, 1.354] | * 1.078 [0.383, 3.040] 1.857 [0.848, 4.070] 1.840 [0.670, 5.055] 5.367 [1.858, 15.502] | 1.004 [0.679, 1.486] 0.677 [0.504, 0.911] 0.854 [0.691, 1.055] 0.830 [0.611, 1.127] 1.477 [1.012, 2.156] |
| **Disease** **duration** (years) | 0.991 [0.985, 0.997] | 1.021 [0.998, 1.025] | 1.001 [0.993, 1.009] | 1.012 [0.998, 1.026] | 1.018 [0.996, 1.040] | 1.003 [0.998, 1.009] |
| **Subjective perceived disease** **severity** | 1.034 [0.999, 1.071] | 0.969 [0.902, 1.040] | 0.994 [0.948, 1.043] | 0.870 [0.813, 0.930] | 1.005 [0.891, 1.134] | 0.978 [0.944, 1.012] |
| **DLQI** | 1.023 [1.010, 1.037] | 1.019 [0.992, 1.047] | 1.008 [0.990, 1.026] | 1.049 [1.024, 1.074] | 1.023 [0.984, 1.064] | 1.016 [1.003, 1.030] |
| **Happiness** | 0.978 [0.942, 1.016] | 0.976 [0.903, 1.055] | 0.938 [0.891, 0.987] | 0.929 [0.866, 0.996] | 0.808 [0.718, 0.909] | 0.928 [0.894, 0.965] |
| **European region** (ref. Southern)  *Eastern Western Northern* | 0.881 [0.693, 1.120] 0.761 [0.612, 0.945] 0.392 [0.296, 0.518] | 1.337 [0.830, 2.153] 0.624 [0.384, 1.014] 0.985 [0.972, 0.997] | 1.123 [0.784, 1.609] 1.094 [0.790, 1.516] 2.160 [1.524, 3.063] | 0.625 [0.390, 1.002] 1.211 [0.808, 1.817] 0.766 [0.457, 1.286] | 0.652 [0.268, 1.585] 0.951 [0.446, 2.027] 1.623 [0.718, 3.668] | 0.640 [0.498, 0.821] 1.266 [1.014, 1.582] 0.828 [0.633, 1.084] |
| **R^2**^** | 0.12 | 0.12 | 0.11 | 0.14 | 0.18 | 0.21 |

**eating disorders were not observed in patients with alopecia areata,* ***Nagelkerke R Square ranges from 0 to 1, with values closer to 1 indicating a better fit of the model. aOR, adjusted odds ratio; CI, confidence interval; DLQI, Dermatological Quality of Life Index*
